# Assessment of Factors Associated With Mental Well-Being Among Chinese Youths at Individual, School, and Province Levels

**DOI:** 10.1001/jamanetworkopen.2023.24025

**Published:** 2023-07-18

**Authors:** Peng Zhang, Fan Yang, Ning Huang, Wei Yan, Bo Zhang, Cancan Zhang, Kaiping Peng, Jing Guo

**Affiliations:** 1 Department of Psychology, Tsinghua University, Beijing, China; 2 Department of Health Policy and Management, School of Public Health, Peking University, Beijing, China; 3 Department of Neurology, Boston Children’s Hospital, Boston, Massachusetts; 4 Division of General Medicine, Beth Israel Deaconess Medical Center, Harvard Medical School, Boston, Massachusetts

## Abstract

**Importance:**

Although rapid urbanization in China provided improved economic prosperity and educational opportunities, it was associated with increased internalizing and externalizing problems among youths, with negative outcomes for mental well-being. Previous studies suggested that factors in individual, school, and regional levels were associated with mental well-being, and comprehensively examining these factors may provide evidence for policies for improving youth mental health.

**Objective:**

To investigate factors associated with mental well-being at individual, school, and province levels among Chinese youths.

**Design, Setting, and Participants:**

This cross-sectional study analyzed nationally representative data collected December 1, 2021, to January 1, 2022, from schools in China. Among 435 schools, 144 institutions had primary school students, 107 institutions had middle school students, 95 institutions had primary and middle school students, and 89 institutions had high school students. A total of 398 520 students were included.

**Exposures:**

Characteristics at individual, school, and province levels.

**Main outcomes and measures:**

The main outcome was mental well-being, assessed by the Short Warwick-Edinburgh Mental Well-being Scale. Linear mixed-effects models were used to investigate individual, school, and regional disparities in mental well-being among youths.

**Results:**

There were 398 520 participants (194 460 females [48.80%]; mean [SD; range] age, 13.78 [2.40; 9-20] years). At the school level, 352 443 students (88.44%) were in public school and 291 354 students (73.11%) were in urban schools. Drinking (coefficient = −1.08; 95% CI, −1.12 to −1.05; *P* < .001), smoking (coefficient = −0.89; 95% CI, −0.96 to −0.83; *P* < .001), and increased age (coefficient per 1-year increase in age = −0.02; 95% CI, −0.03 to −0.01; *P* < .001) were associated with worse mental well-being. Male sex (coefficient = 1.01; 95% CI, 0.98 to 1.04; *P* < .001), being in a 1-child family (coefficient = 0.17; 95% CI, 0.13 to 0.20; *P* < .001), being in the Han group (coefficient vs other ethnic groups = 0.20; 95% CI, 0.14 to 0.26; *P* < .001), a higher frequency and coverage of psychological courses (eg, ≥2/wk for all students vs none: coefficient = 1.02; 95% CI, 0.36 to 1.69; *P* = .003), and a higher level of self-rated popularity in school (coefficient per 1-unit increase in score = 0.89; 95% CI, 0.88 to 0.89; *P* < .001) were associated with improved mental well-being. However, age (eg, age and 1 courses/2 wk for all students: coefficient per 1-year increase in age = −0.047; 95% CI, −0.089 to −0.005; *P* = .03) and sex (eg, female sex and ≥2 courses/wk for some students: coefficient = −0.184; 95% CI, −0.323 to −0.046; *P* = .009) had interactions with the association between psychological courses and mental well-being.

**Conclusions and relevance:**

This study found that social disparities in mental well-being existed and that various factors at different levels were associated with mental well-being among Chinese youths. These findings suggest that public mental health programs may be recommended for associated improvements in regional disparities in mental health resources.

## Key Points

**Question:**

What factors are associated with mental well-being at individual, school, and province levels among youths in China?

**Findings:**

This cross-sectional study of 398 520 youths in China found that some individual-level (eg, younger age and male sex) and school-level (eg, higher frequency and coverage of psychological courses) factors were associated with improved mental well-being. Age and sex had interactions with the association between psychological courses and mental well-being.

**Meaning:**

These findings suggest that public mental health programs may be recommended for associated improvements in youth mental well-being and reductions in regional disparities in youth mental well-being.

## Teaser

This cross-sectional study investigates the association of individual-, school-, and province-level factors with mental well-being among youths in China.

## Introduction

Mental well-being encompasses dimensions of hedonic and eudemonic well-being.^1^ Studies have shown a lower level of mental well-being among youths over time.^2^ Although rapid urbanization in China provided better economic prosperity and educational opportunities,^3^ it was associated with increased internalizing and externalizing problems among youths.^4,5^ Mental well-being has been found to be associated not only with improved health outcomes,^6,7^ but also with better educational outcomes in adolescence and better occupational functioning in adulthood.^8,9^ Upon recognizing the importance of mental well-being, the Chinese government proposed the Healthy China Action Plan to promote child and adolescent health and well-being.^10^ To support the achievement of this proposal, studying factors associated with mental well-being among Chinese youths may be warranted.

The Dahlgren-Whitehead model of social determinants, also known as the rainbow model,^11^ specified factors associated with health inequity on multiple hierarchical levels and suggested that these factors may interact within and between levels. According to this model, various factors at individual, family, and school levels may be associated with youth mental well-being. At the individual level, basic demographic characteristics (ie, age, sex, and body mass index [BMI; calculated as weight in kilograms divided by height in meters squared] level),^12^ self-rated popularity in school,^13^ family socioeconomic status (SES),^14^ and lifestyle (ie, drinking, smoking, and exercise habits)^15^ may be associated with mental well-being. Similarly, the Bronfenbrenner ecological systems theory emphasized the association of levels of factors with healthy outcomes (eg, mental well-being) among individuals.^16^

School, considered as a second home of youths, may be associated with improved mental well-being via a sense of integration.^17^ Mental health programs in school settings play a crucial role in prevention efforts,^18^ and many studies have shown that school-based interventions were associated with improved mental well-being among youths.^19,20^ However, the association between school-provided psychological resources and student mental health has been less studied and remains a topic of debate.^21^

In addition to school-level factors, regional factors, such as Human Development Index (HDI) scores, have been found to be associated with subjective well-being among youths.^22^ According to the livability theory,^23^ people in a developed region may benefit from better economic prosperity and educational opportunities, which may be associated with improved mental well-being.^3^ Some researchers have found that people in a more-developed society show a better mean level of mental well-being.^24^ However, other studies have shown that a higher level of economic development was not always associated with better mental well-being.^25,26^ These findings suggest that it is crucial to comprehensively examine individual-, school-, and regional-level factors that may be associated with mental well-being among Chinese youths using nationwide data.

Given age differences in social pressure^27^ and sex differences in level of mental well-being,^28,29^ interactions among sex, age, and school settings may exist. Although psychological concerns and related settings (eg, recruitment of psychological teachers in schools) in China have drawn attention in recent years, there is a lack of discussion of the outcomes associated with mental health programs at school, especially from a national perspective.^30,31^ Hence, it may be valuable to promote youth mental health to investigate the interacting associations of school setting, age, and sex with the well-being of youths in China.

To address these gaps, we examined risk and protective factors associated with mental well-being in a nationally representative sample in China. Using the rainbow model, we conducted our analysis at individual, school, and regional levels. Furthermore, we explored interactions of age and sex with the association between school mental health programs and mental well-being.

## Methods

This cross-sectional study was approved by the Ethics Committee Board from the Department of Psychology at Tsinghua University. Online electronic consent forms were obtained from all students, their parents, and their schools. The study followed the Strengthening the Reporting of Observational Studies in Epidemiology (STROBE) reporting guideline to report participants, results, and other information.

### Participants

This study analyzed nationally representative cross-sectional data from China collected from December 1, 2021, to January 1, 2022. Before the formal study, we conducted a pilot study in 4 schools in November 2021, enrolling 2678 participants. Based on this study, we revised our questionnaire and enhanced the standardization of the study procedure. The formal study used convenience and multiple-stage sampling and included 435 schools across 31 provinces and 138 cities in China. Initially, we selected 138 cities from each province to obtain a representative sample. Based on the scale of cities, we randomly selected a different numbers of schools in those cities (ie, for a metropolis like Peking, we selected 8 schools in total). Among 435 schools, 144 institutions had primary school students, 107 institutions had middle school students, 95 institutions had primary and middle school students, and 89 institutions had high school students, with a total of 417 144 participants. School-level data were reported by administration staff in target schools. Inclusion criteria were volunteering to join in the survey, having no currently diagnosed mental or physical condition, and studying at school full time. Exclusion criteria were completing the questionnaire within 150 seconds and failing to pass logic questions. Based on these criteria, 2969 participants were excluded. Because head teachers in some schools were difficult to contact or unwilling to provide school-level data (eg, psychological courses), we excluded 15 655 participants from these schools. These missing data were handled using listwise deletion. The final sample size for this study was 398 520 students from 342 schools (101 schools in eastern China, 84 schools in central China, 133 schools in western China, and 24 schools in northeastern China). We compared demographic variables between the original and final samples (eTable 2 in Supplement 1). Questionnaires were answered on computers by students in school settings under the supervision of their head teachers or psychological teachers, who received 1 day of standard training from the research team.

### Measures

#### Mental Well-Being

Mental well-being was assessed using the Short Warwick-Edinburgh Mental Well-being Scale,^32,33^ which has shown good validity and reliability in different populations.^34,35,36^ The scale consists of 7 items describing student mental well-being status during the past 2 weeks by a 5-point Likert scale. The total possible score ranges from 7 to 35, with higher scores indicating better mental well-being. The Cronbach α of the scale in this study was 0.877.

#### Individual-Level Variables

Individual-level variables included sex (male vs female), age, only child status (yes vs no), ethnicity (Han vs others), BMI, drinking and smoking status, days of exercise, sleep duration (calculated by sleep time and rise time), subjective family SES, self-rated popularity in school, objective family SES, and father’s and mother’s education level. All individual variables were self-reported. Owing to the numerous ethnicities of China and the distribution of the data, we divided ethnicity into 2 groups (Han and others). Smoking was a binary variable, with the question asking “whether you smoked during your lifetime”; drinking was assessed in a similar fashion. Days of exercise was assessed by the question “How many days do you exercise more than 1 hour in a week?” In addition, subjective family SES and popularity in school were self-rated by scores ranging from 0 to 10, with a higher score indicating better subjective family SES and greater popularity in school. The objective family SES was produced with factor analysis based on computer ownership, car ownership, and education levels of parents, which was defined as the highest degree held by either parent.^37^ Father’s and mother’s education levels were divided into 6 categories (primary school, middle school, high school, junior college, undergraduate, and master’s and above). These variables were included according to related studies.^38,39,40,41^

#### School-Level Variables

School-level variables included school identification number, type (official vs private), and region (rural vs urban), along with number of institutions around the school (eg, shopping or educational institutions), mean number of psychology teachers per student, psychological activities held by the school (eg, psychological games), coverage and frequency of psychological courses (eg, knowledge about mental health), and honors won. The number of institutions within 3 km from the school was obtained by Baidu map (a widely used guide application in China). These variables were included based on previous related studies.^18,19,20,21^

#### Regional Variables

Based on a previous study,^42^ the Human Development Index (HDI; assessed by life expectancy, expected years of schooling, and gross national income per capita) of the province of residence was included. A total of 31 provinces were divided into 6 regions for comparisons (eTable 1 in Supplement 1). A detailed description of these variables can be found in eTable 3 in Supplement 1.

### Statistical Analysis

We described characteristics of the sample, including individual-level (eg, sex and age), school-level (eg, school type), and regional-level (eg, HDI of the province of residence) factors, as described previously. Moreover, we tested group differences in mental well-being using analysis of variance for category variables and calculated the Pearson correlation coefficient between continuous variables and scores of mental well-being (eTable 3 in Supplement 1).

Linear mixed-effects models (LMEs) were used to investigate individual, school, and regional disparities among youths in mental well-being. Based on R packages lme4 version 1.1-30 and lmerTest version 3.1-3,^43^ we first computed a basic model (model 0, or the empty model) to investigate whether dependent variables were level 1 (individual-level) or 2 (school-level) variables. The intraclass correlation was 0.0947, which indicated the contribution of school-level factors (variance = 2.75) accounted for 9.47% of the total variance (29.09). We computed a series of models and compared candidate models. We then chose the random-intercept model as our final model (model 2). In addition, we conducted a multivariate linear regression model (model 1) to make a contrast with LME models and assessed the cross-level association in model 3 by adding interactions between individual-level and school-level factors. Based on model 2, we added interactions between age and psychological courses and between sex and psychological courses to model 3. Statistical analyses were computed using R statistical software version 4.2.1 (R Project for Statistical Computing), and the significance level was set as a 2-sided α < 0.05.

Although province-level data accounted for little of the total variance, it was still of great necessity to assess potential differences among 31 provinces. Thus, we conducted a set of spatial analyses to assess differences among provinces through GeoDA software version 1.18.0 (Luc Anselin). First, a spatial autocorrelation analysis method was conducted for analyzing the spatial autocorrelation of mental well-being among provinces, which may be represented by global Moran *I*.^44^ Moran *I* ranges from −1 to 1, with *I* > 0, I *=* 0, and *I* < 0 indicating positive, zero, and negative spatial autocorrelation of mental well-being scores among provinces, respectively. We estimated the spatial aggregation of mental well-being by local spatial autocorrelation analysis commonly characterized by the local indicators of spatial association (LISA) index.^45^ We determined 4 spatial aggregation types (high-high, low-high, high-low, and low-low) and locations through a Moran scatter plot and LISA clustering map.

## Results

There were 398 520 participants (194 460 females [48.80%]; mean [SD; range] age, 13.78 [2.40; 9-20] years; 367 668 Han [92.26%]). Table 1 reports characteristics of the final sample. Overall, the mean (SD) score of mental well-being was 24.65 (5.35). The mean (SD) BMI was 21.06 (5.20). Most participants had a sister or brother (290 838 participants [72.98%]). A minority of students drank alcohol (98 214 [24.64%]) or smoked (26 740 [6.71%]). As for exercise, 157 296 students (39.47%) exercised less than 3 days per week, and 134 724 students (33.81%) took exercise more than 5 days per week. At the school level, 352 443 students (88.44%) were in public school, and 291 354 students (73.11%) were in urban schools. In addition, 39 188 students (9.83%) had not participated psychological activities held by schools, and 46 995 students (11.79%) had not been offered psychological courses in school (Table 1).

**Table 1.** Characteristics of Final Sample

Characteristic	Youths, No. (%) (N = 398 520)
Sex	
Female	194 460 (48.80)
Male	204 060 (51.20)
Age, mean (SD), y	13.78 (2.40)
Ethnic group	
Han	367 668 (92.26)
Other	30 852 (7.74)
BMI, mean (SD)	21.06 (5.20)
1-Child family	
No	290 838 (72.98)
Yes	107 682 (27.02)
Drinking	
No	300 306 (75.36)
Yes	98 214 (24.64)
Smoking	
No	371 780 (93.29)
Yes	26 740 (6.71)
Amount of exercise, d/wk	
≤2	157 296 (39.47)
3-4	106 500 (26.72)
5-7	134 724 (33.81)
Subjective family SES, mean (SD)^a^	6.06 (1.97)
Self-rated popularity in school, mean (SD)^a^	6.49 (2.04)
Objective family SES, mean (SD)^b^	0.01 (0.99)
Mother’s education	
≤High school	295 409 (74.13)
Junior college	46 334 (11.63)
Bachelor’s	46 970 (11.79)
≥Master’s	9807 (2.46)
HDI level of resident province	
Low	70 610 (17.72)
Lower middle	84 802 (21.28)
Upper middle	142 827 (35.84)
High	67 655 (16.98)
Very high	9026 (2.26)
School type	
Private	46 077 (11.56)
Public	352 443 (88.44)
Region of school	
Rural	107 166 (26.89)
Urban	291 354 (73.11)
Locations around school	
Shopping institutions	81.15 (19.98)
Exercise institutions	31.06 (28.28)
Traffic stations	84.76 (25.69)
Natural scenery	10.43 (8.53)
Tourist locations	33.41 (32.56)
Educational institutions	72.35 (31.99)
Psychological activities held by school	
None	39 188 (9.83)
1/y	88 663 (22.25)
≥1/Semester	270 669 (67.92)
Psychological courses offered by school	
None	46 995 (11.79)
1/2 wk for some students	94 017 (23.59)
1/wk for some students	80 523 (20.21)
≥2/wk for some students	24 939 (6.26)
1/2 wk for all students	96 988 (24.34)
1/wk for all students	41 354 (10.38)
≥2/wk for all students	13 704 (3.44)
Honors of school	
None	54 555 (13.69)
Awarded in county level	59 572 (14.95)
Awarded in city level	104 236 (26.16)
Awarded in province level	127 499 (31.99)
Awarded in country level	52 658 (13.21)

Abbreviations: BMI, body mass index (calculated as weight in kilograms divided by height in meters squared); HDI, Human Development Index; SES, socioeconomic status.

^a^ Subjective family SES and popularity in school were self-rated by scores (range, 0-10), with a higher score indicating better subjective family SES or greater popularity in school.

^b^ Objective family SES was produced with factor analysis based on computer ownership, car ownership, and the education level of the parents, defined as the highest degree held by either parent. Score range was −2.30 to 1.62.

The Figure illustrates the spatial distribution of mean scores of mental well-being. The lowest scores of mental well-being (range, 22.768-23.849) were observed in central China, including Yunnan, Guizhou, and Guangxi provinces. However, the highest scores appeared in eastern China (range, 26.010-27.091). Overall, Moran *I* was 0.190 (*P* = .03; 99 999 permutations), indicating a weak but positive spatial aggregation. The provinces with the lowest scores of mental well-being were in south China (Guizhou, Guangxi, and Hunan provinces). Results of spatial autocorrelation for mean scores of mental well-being are presented in the eFigure in Supplement 1.

**Figure.**
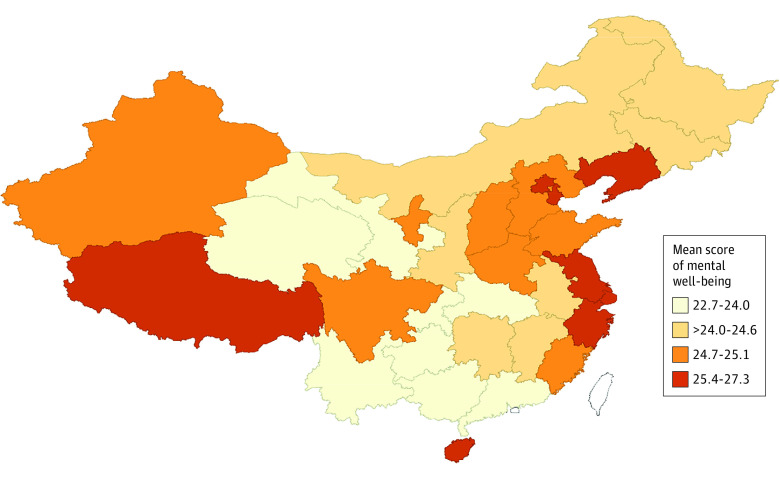
Spatial Distribution of Mean Scores of Mental Well-Being in China by Quartile Blank areas did not have sufficient data.

Table 2 shows the comparison of results between model 1 (multivariate linear regression) and model 2 (LME model). Generally, significant factors were nearly identical in the 2 models except for mother’s education level and HDI score of the province of residence. Mother’s education level of a master’s degree or above compared with high school degree and below (coefficient = 0.10; 95% CI, 0.01 to 0.20; *P* = .04), low HDI (coefficient = 0.52; 95% CI, 0.44 to 0.59; *P* < .001), lower-middle HDI (coefficient = 0.18; 95% CI, 0.11 to 0.26; *P* < .001), and upper-middle HDI (coefficient = 0.36; 95% CI, 0.28 to 0.43; *P* < .001) compared with very low HDI were associated with mental well-being in the multivariate linear model but not the LME model. In both models, higher BMI, drinking (model 1: coefficient = −1.20; 95% CI, −1.24 to −1.17; *P* < .001; model 2: coefficient = −1.08; 95% CI, −1.12 to −1.05; *P* < .001), smoking (model 1: coefficient = −0.95; 95% CI, −1.02 to −0.89; *P* < .001; model 2: coefficient = −0.89; 95% CI, −0.96 to −0.83; *P* < .001), and increased age (model 1: coefficient per 1-year increase in age = −0.04; 95% CI, −0.04 to −0.03; *P* < .001; model 2: coefficient per 1-year increase in age = −0.02; 95% CI, −0.03 to −0.01; *P* < .001) were associated with worse mental well-being. Meanwhile, male sex (model 1: coefficient = 1.00; 95% CI, 0.97 to 1.03; *P* < .001; model 2: coefficient = 1.01; 95% CI, 0.98 to 1.04; *P* < .001), Han ethnicity vs other ethnicities (model 1: coefficient = 0.29; 95% CI, 0.24 to 0.35; *P* < .001; model 2: coefficient vs other ethnic groups = 0.20; 95% CI, 0.14 to 0.26; *P* < .001), being the only child in a family (model 1: coefficient = 0.28; 95% CI, 0.25 to 0.32; *P* < .001; model 2: coefficient = 0.17; 95% CI, 0.13 to 0.20; *P* < .001), higher frequency and coverage of psychological courses (eg, ≥2/wk for all students vs none: model 1: coefficient = 0.87; 95% CI, 0.77 to 0.97; *P* < .001; model 2: coefficient = 1.02; 95% CI, 0.36 to 1.69; *P* = .003), a higher level of self-rated popularity in school (model 1: coefficient per 1-unit increase in score = 0.90; 95% CI, 0.89 to 0.91; *P* < .001; model 2:coefficient per 1-unit increase in score = 0.89; 95% CI, 0.88 to 0.89; *P* < .001), and having greater levels of exercise were associated with better mental well-being. Higher education level of the parents, subjective family SES, and objective family SES were also associated with better mental well-being. In addition, we calculated effect sizes for each variable (eTable 4 in Supplement 1).

**Table 2.** Associations Between Variables and Mental Well-Being

Variable	Model 1^a^		Model 2^b^	
	*B* (95% CI)^c^	*P* value	*B* (95% CI)^c^	*P* value
**Individual level**				
Sex				
Female	0 [Reference]	NA	0 [Reference]	NA
Male	1.00 (0.97 to 1.03)	<.001	1.01 (0.98 to 1.04)	<.001
Age, y^d^	−0.04 (−0.04 to −0.03)	<.001	−0.02 (−0.03 to −0.01)	<.001
Ethnic group				
Han	0.29 (0.24 to 0.35)	<.001	0.20 (0.14 to 0.26)	<.001
Other	0 [Reference]	NA	0 [Reference]	NA
BMI^e^	−0.02 (−0.03 to −0.02)	<.001	−0.02 (−0.03 to −0.02)	<.001
1-Child family				
No	0 [Reference]	NA	0 [Reference]	NA
Yes	0.28 (0.25 to 0.32)	<.001	0.17 (0.13 to 0.20)	<.001
Drinking				
No	0 [Reference]	NA	0 [Reference]	NA
Yes	−1.20 (−1.24 to −1.17)	<.001	−1.08 (−1.12 to −1.05)	<.001
Smoking				
No	0 [Reference]	NA	0 [Reference]	NA
Yes	−0.95 (−1.02 to −0.89)	<.001	−0.89 (−0.96 to −0.83)	<.001
Amount of exercise, d/wk				
≤2	0 [Reference]	NA	0 [Reference]	NA
3-4	0.79 (0.75 to 0.82)	<.001	0.80 (0.77 to 0.84)	<.001
5-7	1.17 (1.43 to 1.50)	<.001	1.49 (1.45 to 1.52)	<.001
Subjective family SES^e^	0.34 (0.33 to 0.35)	<.001	0.33 (0.32 to 0.34)	<.001
Self-rated popularity in school^e^	0.90 (0.89 to 0.91)	<.001	0.89 (0.88 to 0.89)	<.001
Objective family SES^e^	0.10 (0.08 to 0.12)	<.001	0.08 (0.06 to 0.10)	<.001
Mother’s education level				
≤High school	0 [Reference]	NA	0 [Reference]	NA
Junior college	0.20 (0.15 to 0.25)	<.001	0.10 (0.05 to 0.15)	<.001
Bachelor’s	0.29 (0.24 to 0.34)	<.001	0.18 (0.12 to 0.23)	<.001
≥Master’s	0.10 (0.01 to 0.20)	.04	−0.01 (−0.11 to 0.09)	.89
HDI level of resident province				
Very low	0 [Reference]	NA	0 [Reference]	NA
Low	0.52 (0.44 to 0.59)	<.001	0.04 (−0.19 to 0.26)	.75
Lower middle	0.18 (0.11 to 0.26)	<.001	0.06 (−0.21 to 0.33)	.65
Upper middle	0.36 (0.28 to 0.43)	<.001	0.15 (−0.11 to 0.40)	.26
High	0.41 (0.33 to 0.48)	<.001	0.44 (0.17 to 0.70)	.001
Very high	1.13 (1.01 to 1.25)	<.001	0.75 (0.38 to 1.12)	<.001
**School level**				
Type				
Private	0 [Reference]	NA	0 [Reference]	NA
Public	0.41 (0.36 to 0.46)	<.001	0.38 (0.05 to 0.71)	.03
Region				
Rural	0 [Reference]	NA	0 [Reference]	NA
Urban	−0.10 (−0.13 to −0.06)	<.001	−0.01 (−0.29 to 0.27)	.95
Locations around school				
Shopping institutions	0.01 (0.01 to 0.01)	<.001	0.00 (−0.00 to 0.01)	.15
Exercise institutions	0.00 (0.00 to 0.00)	>.99	0.00 (−0.01 to 0.00)	.25
Traffic stations	0.00 (0.00 to 0.00)	<.001	0.00 (−0.00 to 0.01)	.27
Natural scenery	−0.01 (−0.01 to 0.00)	<.001	0.00 (−0.02 to 0.01)	.63
Tourist locations	0.00 (−0.00 to −0.00)	<.001	0.00 (−0.00 to 0.01)	.38
Educational institutions	0.00 (−0.00 to −0.00)	<.001	0.00 (−0.01 to 0.00)	.47
Number of psychology teachers per student, mean^e^	5.64 (−7.72 to 19.00)	.41	8.01 (−37.65 to 53.66)	.73
Psychological activities held by school				
None	0 [Reference]	NA	0 [Reference]	NA
1/y	0.04 (−0.02 to 0.11)	.20	0.25 (−0.17 to 0.67)	.24
≥1/semester	0.18 (0.12 to 0.24)	.10	0.32 (−0.07 to 0.71)	.10
Psychological courses in school				
None				
1/2 wk for some students	0.07 (0.01 to 0.13)	.01	0.04 (−0.38 to 0.46)	.86
1/wk for some students	0.00 (−0.06 to 0.06)	.98	−0.03 (−0.46 to 0.39)	.89
≥ 2/wk for some students	0.53 (0.46 to 0.61)	<.001	0.56 (−0.03 to 1.14)	.06
1/2 wk for all students	0.18 (0.12 to 0.24)	<.001	0.35 (−0.06 to 0.76)	.10
1/wk for all students	−0.04 (−0.11 to 0.03)	.23	0.11 (−0.35 to 0.56)	.65
≥2/wk for all students	0.87 (0.77 to 0.97)	<.001	1.02 (0.36 to 1.69)	.003
Honors of school				
None	0 [Reference]	NA	0 [Reference]	NA
Awarded in county level	−0.10 (−0.16 to −0.04)	<.001	0.00 (−0.02 to 0.03)	.70
Awarded in city level	−0.22 (−0.27 to −0.17)	<.001	−0.01 (−0.04 to 0.02)	.44
Awarded in province level	0.15 (0.09 to 0.20)	<.001	0.02 (−0.01 to 0.04)	.30
Awarded in country level	0.05 (−0.01 to 0.11)	.10	0.01 (−0.02 to 0.03)	.74

Abbreviations: BMI: body mass index (calculated as weight in kilograms divided by height in meters squared); HDI, Human Development Index; NA, not applicable; SES: socioeconomic status.

^a^ Conventional linear regression.

^b^ Linear mixed-effects models.

^c^ *B* represents the unstandardized coefficient.

^d^ Per 1-year increase in age.

^e^ Per 1-unit increase.

Table 3 presents the cross-level interaction of school-level factors and individual-level factors in the association with mental well-being. Age (eg, age and courses once/2 wk for all students: coefficient per 1-year increase in age = −0.047; 95% CI, −0.089 to −0.005; *P* = .03) and sex (eg, female sex and ≥2 courses/wk for some students: coefficient = −0.184; 95% CI, −0.323 to −0.046; *P* = .009) had interactions with psychological courses in the association with mental well-being. These findings suggest that older students may benefit more from taking more psychological courses and male students may benefit less in psychological courses.

**Table 3.** Interactions of School-and Individual-Level Factors in Association With Mental Well-Being^a^

Variable	*B* (95% CI)^b^	*P* value
Sex		
Female	0 [Reference]	NA
Male	0.097 (0.090 to 0.105)	<.001
Ethnic group		
Han	0.010 (0.007 to 0.013)	<.001
Other	0 [Reference]	NA
BMI^c^	−0.023 (−0.026 to −0.020)	<.001
1-Child family		
No	0 [Reference]	NA
Yes	0.014 (0.011 to 0.017)	<.001
Drinking		
No	0 [Reference]	NA
Yes	−0.087 (−0.090 to −0.084)	<.001
Smoking		
No	0 [Reference]	NA
Yes	−0.042 (−0.045 to −0.039)	<.001
Amount of exercise, d/wk		
≤2	0 [Reference]	NA
3-4	0.066 (0.063 to 0.069)	<.001
5-7	0.131 (0.128 to 0.135)	<.001
Subjective family SES^c^	0.121 (0.118 to 0.125)	<.001
Self-rated popularity in school^c^	0.337 (0.333 to 0.340)	<.001
Objective family SES^c^	0.014 (0.011 to 0.018)	<.001
Mother’s education level		
≤High school	0 [Reference]	NA
Junior college	0.098 (0.049 to 0.146)	<.001
Bachelor’s	0.176 (0.123 to 0.228)	<.001
HDI level of resident province		
Very low	0 [Reference]	NA
High	0.430 (0.166 to 0.695)	.001
Very high	0.749 (0.381 to 1.116)	<.001
School type		
Private	0 [Reference]	NA
Public	0.376 (0.044 to 0.708)	.03
Psychological courses offered		
None	0 [Reference]	NA
1/2 wk For all students	1.026 (0.332 to 1.721)	.004
Age: ≥2 wk for some students	0.055 (0.001 to 0.109)	.05
Age: 1/2 wk for all students	−0.047 (−0.089 to −0.005)	.03
Female: ≥2/wk for some students	−0.184 (−0.323 to −0.046)	.009
Female: 1/2 wk for all students	−0.118 (−0.218 to −0.019)	.02
Female: ≥2/wk for all students	−0.288 (−0.460 to −0.116)	.001

Abbreviations: BMI, body mass index (calculated as weight in kilograms divided by height in meters squared); HDI, Human Development Index; NA, not applicable; SES, socioeconomic status.

^a^ Based on model 2 (linear mixed model), interactions between age, sex, and psychological courses into model 3. Only statistically significant results of model 3 are presented in the table.

^b^ *B* represents the unstandardized coefficient.

^c^ Per 1-unit increase.

## Discussion

This cross-sectional study analyzed a large nationally representative data set to investigate the association of individual-, school-, and regional-level factors with mental well-being among Chinese youths. For individual-level factors, sex, age, BMI, drinking and smoking status, being in a 1 child family, exercise level, family SES, popularity in school, and mother’s education level were associated with mental well-being. Our study found that females had worse mental health than males. This is consistent with previous studies, which found that females had worse mental health than males, which was associated with sensitivity to emotional changes^28^ and experiencing more restricted gender roles and body dissatisfaction.^29^ Similarly, older youths had worse mental health status compared with younger individuals,^12^ which may be explained by more life stress (ie, higher schoolwork pressure) among older youths.^27^ Youths from a 1-child family had better mental well-being; this finding is in line with the theory of resource dilution, which states that in China, a child in a 1-child family receives more attention from parents and has more family resources.^46,47^ In turn, the sense of security and confidence,^47^ which are associated with better a mental health condition, may be enhanced. Unhealthy lifestyles (eg, drinking, smoking, and doing little exercise) may be risk factors associated with adverse mental health outcomes.^15^ In addition, better family SES may bring more family social capital, which may be associated with improved mental well-being.^48^ Better self-rated popularity in high school may indicate better perceived social capital (ie, social support) in school, which has been associated with better mental health.^13^ In addition, previous researchers found that parental educational attainment was positively associated with mental well-being,^40^ and this is in accordance with our findings.

At the school-level, school type and psychological courses held by the school were associated with the mental well-being of students. Individuals in public schools had better mental health outcomes. This may be explained by the stricter regulations among private vs public schools,^49^ and this may be associated with a series of negative outcomes, including worse mental well-being. Regardless of school type, the coverage and frequency of psychological courses held by a school were associated with mental well-being, which is in line with findings from previous studies.^50,51^ There is no doubt that psychological education may be associated with improved knowledge of psychology among students, which is valuable for people in handling mental problems. However, we found that only psychological courses twice a week or more for all students had significant outcomes. This finding suggests that both the coverage and frequency of courses may be associated with health outcomes (ie, mental well-being).

Analyses of interactions between 2 levels showed that psychological courses were associated with less improvement in mental well-being among males. Male students may be less focused on the school coursework, or they may have had better mental well-being status.^28,29^ These findings suggest that it may be more difficult to improve mental well-being in male students, and these factors may explain why male students experienced less benefit from psychological courses. Regarding interactions with age, older students may receive better feedback than younger students. This may be explained by the greater social pressure (ie, school pressure) among older students mentioned prevously^27^ and worse mental health status,^12^ which may be associated with greater focus on psychological courses.

Among regional factors, HDI level was associated with student mental well-being. Regions with high HDI regions are known to have greater levels of social resources and medical care, better educational opportunities, and greater economic prosperity,^3^ contributing to better living conditions and better mental health.^23^ Although our main analysis focused on individual- and school-level factors associated with mental well-being, the spatial analysis found a positive spatial aggregation, and the lowest scores of mental well-being were commonly observed in central China, including Yunnan, Guizhou, and Guangxi provinces.

These findings suggest several recommendations for policy makers that may support achieving goals of the Healthy China 2030 Plan. First, increasing access to mental health resources, such as after-school programs that promote physical activity, and support for educational and career opportunities that enhance SES may be recommended. Second, psychological courses provided to enhance mental well-being should be offered frequently, have wide coverage, and be tailored to needs of students based on age and sex. Third, addressing regional disparities in access to mental resources by investing in education and infrastructure may be associated with improved youth mental well-being in less-developed regions.

### Limitations

There are several limitations in the study. First, cross-sectional data cannot be used to investigate causal relationships, and there are slight differences between the origin sample and the final sample. Second, weak spatial aggregation warrants further examination. Third, this study did not account for previous mental health problems of students owing to data limitations, which may have introduced bias. Fourth, this study was based on a questionnaire with self-reported data, and some variables (eg, days of exercise) may not be assessed by a well-developed scale. Similarly, individuals with lower levels of mental well-being may have a greater tendency to not report. However, the large sample size may balance this effect to some extent. Fifth, because some schools indicated that COVID-19–related items may bring stress to students, we did not include these items in our survey, which made us unable to discuss the potential contribution of the COVID-19 pandemic to mental well-being. The COVID-19 pandemic has been reported to have associations with youth mental well-being.^52,53^ Sixth, owing to the large size of the sample, variables could more easily attain statistical significance. Thus, we calculated effect sizes for each variable to report more details. Despite these limitations, our study shed light on student mental well-being at individual, school, and provincial levels.

## Conclusions

This cross-sectional study found that social disparities existed and that various factors in different levels were associated with student mental well-being. These findings suggest that public mental health programs should be addressed to improve regional disparities in mental health resources and that age and sex should be especially considered when schools carry out psychological courses to enhance student mental well-being.

## Notes

Supplement 1. **eTable 1.** Human Development Index (HDI) of Province of Residence **eTable 2.** Comparison Between Original and Final Samples **eTable 3.** Details About Variables **eTable 4.** Group Differences in Mental Well-Being Among Variables **eFigure.** Spatial Autocorrelation for Mean Scores of Mental Well-Being

Supplement 2.
Data Sharing Statement
