# Supplementary material for: Assessment of Factors Associated With Mental Well-Being Among Chinese Youths at Individual, School, and Province Levels
Source: JAMA Netw Open. 2023 Jul 18;6(7):e2324025. doi: 10.1001/jamanetworkopen.2023.24025 (PMC10354672; doi:10.1001/jamanetworkopen.2023.24025)
Supplement: Supplement 1. — eTable 1. Human Development Index (HDI) of Province of Residence eTable 2. Comparison Between Original and Final Samples eTable 3. Details About Variables eTable 4. Group Differences in Mental Well-Being Among Variables eFigure. Spatial Autocorrelation for Mean Scores of Mental Well-Being [file jamanetwopen-e2324025-s001.pdf]

Supplemental Online Content

Zhang P, Yang F, Huang N, et al. Assessment of factors associated with mental well-being among Chinese youths at individual, school, and province levels. *JAMA Netw Open*. 2023;6(7):e2324025. doi:10.1001/jamanetworkopen.2023.24025

eTable 1. Human Development Index (HDI) of Province of Residence

eTable 2. Comparison Between Original and Final Samples

eTable 3. Details About Variables

eTable 4. Group Differences in Mental Well-Being Among Variables

eFigure. Spatial Autocorrelation for Mean Scores of Mental Well-Being

This supplemental material has been provided by the authors to give readers additional information about their work.

© 2023 Zhang P et al. *JAMA Network Open.*

eTable 1. Human Development Index (HDI) of Resident Province

**Percentile of the HDI**	**HDI thresholds**	**Province**
< 10th	<0·667	Guizhou, Qinghai, Xizang, and Yunnan
10th to 25th	(0·667, 0·708]	Anhui, Gansu, Guangxi, and Sichuan
25th to 50th	(0·708, 0·733]	Hainan, Hebei, Henan, Heilongjiang, Jiangxi, Ningxia, Shanxi, and Xinjiang
50th to 70th	(0·733, 0·754]	Fujian, Hubei, Hunan, Jilin, Inner-Mongolia, Shandong, Shannxi, and Chongqing
75th to 90th	(0·754, 0·784]	Guangdong, Jiangsu, Liaoning, and Zhejiang
> 90th	> 0·784	Beijing, Shanghai, and Tianjin

© 2023 Zhang P et al. *JAMA Network Open.*

eTable 2. Comparison between origin sample and final sample.

Variables	Levels	Selected Sample (N = 398520)	Origin Sample (N = 414175)	*P*-value
		**Mean ± SD/ N (%)**	**Mean ± SD/ N (%)**	
Sex	Female	194460 (48.80%)	202023 (48.78%)	0.870
	Male	204060 (51.20%)	212152 (51.22%)	
Age, years	Continuous	13.78 ± 2.40	13.71 ± 2.41	< 0.001
Race	Other	30852 (7.74%)	32580 (7.87%)	0.037
	Han	367668 (92.26%)	381595 (92.13%)	
BMI	Continuous	21.06 ± 5.20	21.04 ± 5.24	0.238
Only one child	NO	290838 (72.98%)	302644 (73.07%)	0.352
	Yes	107682 (27.02%	111531 (26.93%)	
Drinking	NO	300306 (75.36%)	313752 (75.75%)	< 0.001
	Yes	98214 (24.64%)	100423 (24.25%)	
Smoking	NO	371780 (93.29%)	386997 (93.44%)	0.008
	Yes	26740 (6.71%)	27178 (6.56%)	
Days of exercise, day(s)	≤ 2 per week	157296 (39.47%)	162284 (39.18%)	0.028
	3 - 4 per week	106500 (26.72%)	111091 (26.82%)	
	5 - 7 per week	134724 (33.81%)	140800 (34.00%)	
Subjective family SES	Continuous	6.06 ± 1.97	6.08 ± 1.98	< 0.001
Self-rated popularity in school	Continuous	6.49 ± 2.04	6.50 ± 2.04	0.012

© 2023 Zhang P et al. *JAMA Network Open.*

Objective family SES	Continuous	0.01 ± 0.99	-0.00 ± 1.00	0.002
Mother's education	High school and below	295409 (74.13%)	307172 (74.16%)	0.218
	Junior college	46334 (11.63%)	47741 (11.53%)	
	Bachelor	46970 (11.79%)	48834 (11.79%)	
	Master and above	9807 (2.46%)	10428 (2.52%)	
HDI of resident province	Very low HDI	23600 (5.92%)	25157 (6.07%)	< 0.001
	Low HDI	70610 (17.72%)	78127 (18.86%)	
	Lower-middle HDI	84802 (21.28%)	87690 (21.17%)	
	Upper-middle HDI	142827 (35.84%)	144126 (34.80%)	
	High HDI	67655 (16.98%)	69730 (16.84%)	
	Very high HDI	9026 (2.26%)	9345 (2.26%)	
School type	Private	46077 (11.56%)	48498 (11.71%)	0.038
	Public	352443 (88.44%)	365677 (88.29%)	
Region of school	Rural	107166 (26.89%)	115920 (27.99%)	< 0.001
	Urban	291354 (73.11%)	298255 (72.01%)	

© 2023 Zhang P et al. *JAMA Network Open.*

eTable 3. Details about the variables.

Variables	Levels	Type	Assessment	Note
Mental well-being	Continuous	Ratio	SWEMWBS	/
Sex	Female<br>Male	Nominal	Self-reported	/
Age, years	Continuous	Interval	Self-reported	/
Race	Other<br>Han	Nominal	Self-reported	/
BMI	Continuous	Ratio	Self-reported	Based on self-reported height and weight
Only one child	NO<br>Yes	Nominal	Self-reported	/
Drinking	NO<br>Yes	Nominal	Self-reported	"whether you drank during your life time'
Smoking	NO<br>Yes	Nominal	Self-reported	"whether you smoked during your life time'
Days of exercise, day(s)	≤ 2 per week<br>3 - 4 per week<br>5 - 7 per week	Ordinal	Self-reported	"how many days do you exercise more than 1 hour in a week"
Subjective family SES	Continuous	Interval	Self-reported	ranging from 0 to 10
Self-rated popularity in school	Continuous	Interval	Self-reported	ranging from 0 to 10
Objective family SES	Continuous	Ratio	Self-reported	produced with factor analysis based on computer ownership, car ownership, and the education level of the parents
Mother's education	High school and below<br>Junior college	Ordinal	Self-reported	/

© 2023 Zhang P et al. *JAMA Network Open.*

	Bachelor<br>Master and above			
HDI of resident province	Very low HDI<br>Low HDI<br>Lower-middle HDI<br>Upper-middle HDI<br>High HDI<br>Very high HDI	Ordinal	Previous research based	assessed by the health, education and the income level of each province
School type	Private<br>Official	Nominal	Self-reported	/
Region of school	Rural<br>Urban	Nominal	Self-reported	/
Shopping institutions around school	Continuous			
Exercise institutions around school	Continuous			
Traffic stations around school	Continuous			
Natural sceneries around school	Continuous	Interval	Calculated	obtained by Baidu map (a widely used guide application in China)
Tourist locations around school	Continuous			
Education institutions around school	Continuous			
Psychological activities held by school	None<br>1/year<br>≥ 1/semester	Ordinal	Self-reported	i.e., psychological games

© 2023 Zhang P et al. *JAMA Network Open.*

Psychological courses	None<br>1/two weeks for part of students<br>1/week for part of students<br>≥ 2/week for part of students<br>1/two weeks for all students<br>1/week for all students<br>≥ 2/week for all students	Ordinal	Self-reported	i.e., knowledge about mental health
Honors of school	None<br>Awarded in county level<br>Awarded in city level<br>Awarded in province level<br>Awarded in country level	Ordinal	Self-reported	i.e., rewarded by the city government

© 2023 Zhang P et al. *JAMA Network Open.*

eTable 4. Group differences of menta well-being among variables.

Variables		Mean (SD)/ N (%)	*P*-value	Effect size (η²/r²)
Sex				
	Female	194460 (48.80)	< 0.001	0.013
	Male	204060 (51.20)		
Age, y		13.78 (2.40)	< 0.001	0.027
Race				
	Other	30852 (7.74)	< 0.001	0.001
	Han	367668 (92.26)		
BMI		21.06 (5.20)	< 0.001	0.003
Only one child				
	NO	290838 (72.98)	< 0.001	0.006
	Yes	107682 (27.02)		
Drinking				
	NO	300306 (75.36)	< 0.001	0.032
	Yes	98214 (24.64)		
Smoking				
	NO	371780 (93.29)	< 0.001	0.016
	Yes	26740 (6.71)		
Days of exercise, day(s)				
	≤ 2 per week	157296 (39.47)		
	3 - 4 per week	106500 (26.72)	< 0.001	0.016
	5 - 7 per week	134724 (33.81)		
Subjective family SES		6.06 (1.97)	< 0.001	0.133
Self-rated popularity in school		6.49 (2.04)	< 0.001	0.214

© 2023 Zhang P et al. *JAMA Network Open.*

Objective family SES	0.01 (0.99)	< 0.001	0.027
Mother's education			
High school and below	295409 (74.13)	< 0.001	0.022
Junior college	46334 (11.63)		
Bachelor	46970 (11.79)		
Master and above	9807 (2.46)		
HDI of resident province			
Low HDI	70610 (17.72)	< 0.001	0.006
Lower-middle HDI	84802 (21.28)		
Upper-middle HDI	142827 (35.84)		
High HDI	67655 (16.98)		
Very high HDI	9026 (2.26)		
Region of school			
Rural	107166 (26.89)	< 0.001	0.006
Urban	291354 (73.11)		
Shopping institutions around school	81.15 (19.98)	< 0.001	0.002
Exercise institutions around school	31.06 (28.28)	< 0.001	0.004
Traffic stations around school	84.76 (25.69)	< 0.001	0.002
Natural sceneries around school	10.43 (8.53)	< 0.001	0.001
Tourist locations around school	33.41 (32.56)	< 0.001	0.003
Education institutions around school	72.35 (31.99)	< 0.001	0.003
Psychological activities held by school			
None	39188 (9.83)	< 0.001	0.001
1/year	88663 (22.25)		

© 2023 Zhang P et al. *JAMA Network Open.*

≥ 1/semester	270669 (67.92)		
Psychological courses			
None	46995 (11.79)		
1/two weeks for part of students	94017 (23.59)		
1/week for part of students	80523 (20.21)		
≥ 2/week for part of students	24939 (6.26)	< 0.001	0.004
1/two weeks for all students	96988 (24.34)		
1/week for all students	41354 (10.38)		
≥ 2/week for all students	13704 (3.44)		
Honors of school			
None	54555 (13.69)		
Awarded in county level	59572 (14.95)		
Awarded in city level	104236 (26.16)	< 0.001	0.002
Awarded in province level	127499 (31.99)		
Awarded in country level	52658 (13.21)		

© 2023 Zhang P et al. *JAMA Network Open.*

a

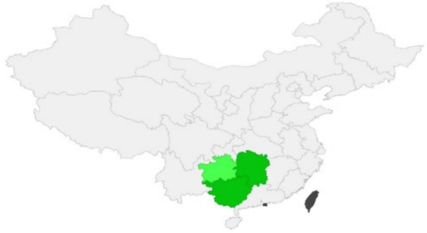

b

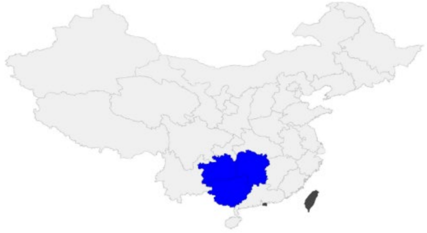

eFigure. Spatial auto-correlation for mean scores of mental well-being in China. a. The blank areas indicate no statistical significance, light green indicates *p*-value < 0.05, green indicates *p*-value < 0.01 and the black indicates undefined due to data insufficient. b. The blank areas indicate no statistical significance, blue indicates Low-Low spatial aggregation and the black indicates undefined due to data insufficient.

© 2023 Zhang P et al. *JAMA Network Open.*
