# Supplementary material for: Assessment of Factors Associated With Mental Well-Being Among Chinese Youths at Individual, School, and Province Levels
Source: JAMA Netw Open. 2023 Jul 18;6(7):e2324025. doi: 10.1001/jamanetworkopen.2023.24025 (PMC10354672; doi:10.1001/jamanetworkopen.2023.24025)
Supplement: Supplement 2. — Data Sharing Statement [file jamanetwopen-e2324025-s002.pdf]

Data Sharing Statement

Zhang. Assessment of Factors Associated With Mental Well-Being Among Chinese Youths at Individual, School, and Province Levels. *JAMA Netw Open*. Published online July 18, 2023. doi:10.1001/jamanetworkopen.2023.24025

Data
Data available: No
